# Social Risk Adjustment and Bonus Eligibility in Medicare Advantage Star Ratings

**DOI:** 10.1001/jamahealthforum.2026.0326

**Published:** 2026-03-27

**Authors:** Andrew Anderson, Rajesh Satpathy-Horton, Mark K. Meiselbach

**Affiliations:** 1 Department of Health Policy and Management, Johns Hopkins Bloomberg School of Public Health, Baltimore, Maryland

## Abstract

**Importance:**

The Medicare Advantage (MA) star ratings program determines eligibility for quality bonus payments that directly affect plan revenue, benefit generosity, and market competitiveness. To improve fairness, the Centers for Medicare & Medicaid Services (CMS) introduced the Categorical Adjustment Index (CAI) in 2017, which adjusts ratings based on enrollee social risk factors. How these adjustments have affected bonus eligibility for MA contracts over time remains unclear.

**Objective:**

To assess the association between CAI adjustments and bonus eligibility in the MA star ratings program and assess whether the policy supports fair comparisons in performance measurement.

**Design, Setting, and Participants:**

This retrospective cohort panel study included MA contracts with CMS star ratings data from 2014 to 2025, including at least 1 year before and after CAI implementation. Publicly available CMS files were used to merge contract-level CAI values and star ratings for overall, Part C, and Part D domains.

**Exposures:**

Annual CAI value (range: −0.10 to 0.20), assigned to MA contracts based on its proportion of beneficiaries who are dually eligible for Medicare and Medicaid or receive a low-income subsidy and those with disability status.

**Main Outcomes and Measures:**

Bonus eligibility, defined as an overall, Part C, or Part D star rating of 4.0 or higher. Fixed-effects linear regression models were used to assess the association between CAI value and bonus eligibility.

**Results:**

Of 339 contracts between 2017 and 2025, 156 (46.0%) became bonus eligible at least once due to CAI adjustments, leading to 398 CAI-based bonus eligibility transitions. Each 0.10-point increase in CAI value was associated with a 0.16-point increase in bonus eligibility probability (β = 1.41; 95% CI, 0.64–2.18; *P* < .001). Contracts with the highest CAI values remained less likely to achieve bonus eligibility even after adjustment.

**Conclusions and Relevance:**

The results of this study provide evidence on the potential role of CAI in MA contract performance; future research should assess whether alternative adjustment methods can create better comparisons of performance between MA contracts.

## Key Points

**Question:**

Did Medicare Advantage (MA) contract bonus eligibility change following the Centers for Medicare & Medicaid Services’ implementation of social risk adjustment?

**Findings:**

In this cohort study of 339 Medicare Advantage contracts observed from 2014 to 2025, 46.0% became eligible for quality bonus payments in at least 1 year due to Categorical Adjustment Index (CAI) adjustments. Each 0.10-point increase in the CAI was associated with a 0.16-point higher probability of bonus eligibility.

**Meaning:**

The results of this study provide evidence on the potential role of CAI in MA contract performance; future research should assess whether alternative adjustment methods can create better comparisons of performance between MA contracts.

## Teaser

This cohort study quantifies how many contracts became eligible for quality bonuses due to Categorical Adjustment Index (CAI) adjustments and estimates the association between CAI values, adjusted star ratings, and bonus status in Medicare Advantage contracts between 2014 and 2025.

## Introduction

Medicare Advantage (MA) star ratings play an important role in determining quality bonus payments, shaping plan revenue, beneficiary benefits, and market competitiveness.^1,2,3^ Since 2012, MA contracts earning 4 or more of 5 stars have qualified for bonus payments from the Centers for Medicare & Medicaid Services (CMS), creating incentives for plans to achieve high scores.^4^ However, there are long-standing concerns about the fairness of these ratings for plans serving a high proportion of beneficiaries with social risk factors, particularly recipients in the low-income subsidy (LIS) program, enrollees with disabilities as well as those who are dual eligible for Medicare and Medicaid.^5,6,7^ Contracts serving higher shares of these populations have historically received lower star ratings, raising the risk of inequitable bonus distribution and unintended consequences for health plans serving populations with high levels of need.^8^

In response, CMS implemented the Categorical Adjustment Index (CAI) in 2017 to account for differences in enrollee characteristics that may affect performance in quality measures but lie outside of plan control.^9^ Plans disproportionately serving populations at high social risk could be disadvantaged in star ratings, creating potential disincentives for enrolling these beneficiaries. The CAI assigns contract-level adjustments to star ratings based on the proportion of enrollees with LIS and disability status in each plan, using a stratified approach that adds or subtracts points from a contract’s overall, Part C, and Part D summary scores.^10^ Star ratings are based on rounded summary scores where many contracts cluster just below or above the 4-star threshold, making small adjustments such as the CAI especially consequential for determining bonus eligibility. As a result, the CAI then pushes some contracts above the 4-star threshold, making them newly eligible for quality bonus payments.^11^

Yet, it remains unknown how the CAI has affected star ratings and bonus eligibility for MA plans over time. While CAI adjustments may raise ratings for contracts serving enrollees with disadvantages, they may still fall short of creating fair comparisons across plans in the star ratings program.^12^ This study addresses that gap through a cohort consisting of a retrospective panel of MA contracts from 2014 to 2025 to assess CAI values and bonus eligibility. We quantify how many contracts became eligible for quality bonuses due to CAI adjustments and estimate the association between CAI values, adjusted star ratings, and bonus status. Our findings provide new evidence on the role of CAI in shaping MA contract performance over time.

## Methods

In this cohort study, we conducted a retrospective panel analysis of MA contracts using publicly available data from the CMS star ratings files from 2014 to 2025. We combined contract-level star ratings with Final Adjustment Category data from the CMS CAI files, which report CAI values assigned to MA contracts based on enrollee disability, LIS status, and dual eligibility.^13,14^ This study was deemed exempt by the Johns Hopkins Bloomberg School of Health Institutional Review Board as it did not involve human participants research.

### Study Sample

The participant flow diagram is depicted in eFigure 1 of Supplement 1. We included MA contracts with at least 1 year of data both before (2014–2016) and after (2017–2025) the implementation of the CAI, enabling within-contract comparisons over time. This restriction allows for capturing movement into bonus eligibility attributable to CAI adjustments. Separate analytic samples were constructed for overall, Part C, and Part D star ratings. Contracts were required to have a reported (CAI-adjusted) star rating and an assigned CAI value, which allowed us to derive the corresponding unadjusted rating. The final analytic samples included 339 contracts for overall ratings, 345 for Part C, and 426 for Part D.

### Measures

The primary outcome was bonus eligibility, defined as achieving a star rating of 4.0 or higher in the overall, Part C, or Part D domain. This threshold determines whether an MA contract qualifies for quality bonus payments. We assessed this outcome in 2 ways: as a binary indicator of whether a contract was bonus-eligible in a given year and as a within-year transition outcome, defined as crossing the 4-star threshold only after the CAI was applied.

The primary independent variable was the CAI value, which ranged from –0.10 to 0.20 applied at the contract score level beginning in 2017. CAI values are assigned at the contract level based on relative enrollee composition, with negative values applied to contracts with lower proportions of beneficiaries with LIS, dual eligibility, or disabilities and positive values applied to those with higher proportions, thereby lowering or raising scores accordingly. CMS only publishes the rounded, CAI-adjusted ratings, so we approximated unadjusted ratings by subtracting the CAI value in the contract from the published rounded rating. These values are therefore not official CMS ratings but provide a reasonable proxy for assessing how CAI affects bonus eligibility.

We included several contract characteristics as covariates: contract age in years, total enrollment (modeled as the natural log), the percentage of enrollees receiving an LIS in Part D, the percentage enrolled in a Special Needs Plan (SNP), and the primary geographic region of the contract (Northeast, Midwest, South, West, or National). All models also included measurement year indicators, with 2016 as the reference year, to account for secular trends and policy changes.

### Statistical Analysis

We first quantified the number and proportion of MA contracts that became bonus eligible due to CAI adjustments in each domain (overall, Part C, and Part D) between 2017 and 2025. Contract characteristics were summarized by whether a contract never crossed the 4-star bonus threshold and within the first year of becoming bonus eligible due to CAI. Associations between CAI values and bonus eligibility were estimated using linear fixed-effects panel regression models with SEs clustered at the contract level. Models included contract fixed effects to account for time-invariant differences and year fixed effects (reference, 2016) to account for secular trends and policy changes. The primary model (by-year model) included all contract-year observations and evaluated the association between annual CAI value and bonus eligibility status. A secondary specification (within-year transition model) limited the sample to contract-years in which the unadjusted rating fell below the bonus threshold and modeled the probability of crossing the 4-star cutoff after applying the CAI. We also grouped contracts into deciles of average CAI value (decile 1 = lowest social risk; decile 10 = highest), and within each decile, we calculated the percentage of contract-years eligible for bonus payments before and after CAI adjustment. As a supplemental analysis, we estimated fixed-effects regressions using the continuous star rating (0–5 scale) as the outcome to assess the association between CAI values and absolute rating levels. Statistical significance was defined as 2-sided *P* < .05. Analyses were conducted using Stata version 18.0 (StataCorp).

## Results

From 2017 to 2025, 46.0% of MA contracts (156 of 339) became bonus eligible (≥4 stars) in at least 1 year due to CAI adjustments in the overall rating domain, resulting in 398 total transitions to bonus eligibility of 2691 contract-year observations. In the Part D domain, 61.5% of contracts (262 of 426) experienced at least 1 CAI-based transition (610 of 3340 events) compared with 36.8% (127 of 345) in the Part C domain (276 of 2734 events) (Table 1). Post-CAI entrants had modestly higher initial star ratings than pre-CAI entrants (3.6 vs 3.3) (eTable 1 in Supplement 1).

**Table 1.** Medicare Advantage Contracts Gaining Bonus Eligibility Due to CAI, by Domain (2017–2025)^a^

Domain	Total contracts, No.	Contracts ever gaining bonus eligibility via CAI, No. (%)	CAI-based bonus eligibility events, No./total No. (%)
Overall	339	156 (46.0)	398/2691 (14.8)
Part C	345	127 (36.8)	276/2734 (10.1)
Part D	426	262 (61.5)	610/3340 (18.3)

Abbreviation: CAI, Categorical Adjustment Index.

^a^ Percentages reflect the share of CAI-based bonus eligibility events of all contract-year observations between 2017–2025 for each domain. Contracts could experience multiple events over time. The sample includes only contracts with at least 1 year of pre-CAI data (2014–2016). Unadjusted ratings are approximated by subtracting the CAI value from the published rounded rating; the Centers for Medicare and Medicaid Services does not release rounded ratings without CAI.

Contracts that became bonus eligible due to CAI differed significantly from those that never did (Table 2). On average, they had higher unadjusted star scores (mean [SD], 4.10 [0.34] vs 3.54 [0.45]; *P* < .001), were older (mean [SD] age, 18.0 [8.2] vs 14.1 [6.8] years; *P* < .001), and had lower mean (SD) percentages of LIS enrollees (21.24% [23.60%] vs 46.73% [34.06%]; *P* < .001) and SNP enrollment (24.40% [30.89%] vs 52.67% [35.92%]; *P* < .001). These contracts also had larger enrollment on average (mean [SD] total enrollees, 100.45 [207.17] thousand vs 36.95 [66.70] thousand; *P* < .001). Regional distribution differed significantly, with bonus-adjusted contracts more often classified as national (52 [15.85%] vs 22 [6.71%]) and less often located in the South (23 [7.01%] vs 46 [14.02%]) (*P* < .001).

**Table 2.** Characteristics of Medicare Advantage Contracts by CAI Bonus Adjustment Status (2017–2025)^a^

Characteristic	Never adjusted to bonus (n = 183)	Adjusted to bonus (n = 156)	*P* value
Unadjusted overall star rating	3.54 (0.45)	4.10 (0.34)	<.001
Contract age, y	14.01 (6.75)	18.02 (8.20)	<.001
SNP enrollment, %	52.67 (35.92)	24.40 (30.89)	<.001
MA-Part D LIS enrollment, %	46.73 (34.06)	21.24 (23.60)	<.001
MA enrollees in thousands	4.29 (21.21)	23.26 (94.53)	.06
Part D enrollees in thousands	34.95 (56.24)	87.63 (166.71)	<.001
Total enrollees in thousands	36.95 (66.70)	100.45 (207.17)	<.001
Primary region, No. (%)			
Northeast	32 (9.76)	20 (6.10)	<.001
Midwest	18 (5.49)	27 (8.23)	
South	46 (14.02)	23 (7.01)	
West	56 (17.07)	32 (9.76)	
National	22 (6.71)	52 (15.85)	

Abbreviations: CAI, Categorical Adjustment Index; LIS, low-income subsidy; MA, Medicare Advantage; SNP, Special Needs Plan.

^a^ Values are presented as mean (SD) unless otherwise indicated. Characteristics for contracts that crossed into bonus eligibility due to the CAI are shown in the year of first crossing; characteristics for contracts that never crossed are shown in 2017. *P* values are from Welch 2-sample *t* tests for continuous variables and Pearson χ^2^ tests for categorical variables.

**Figure 1.**
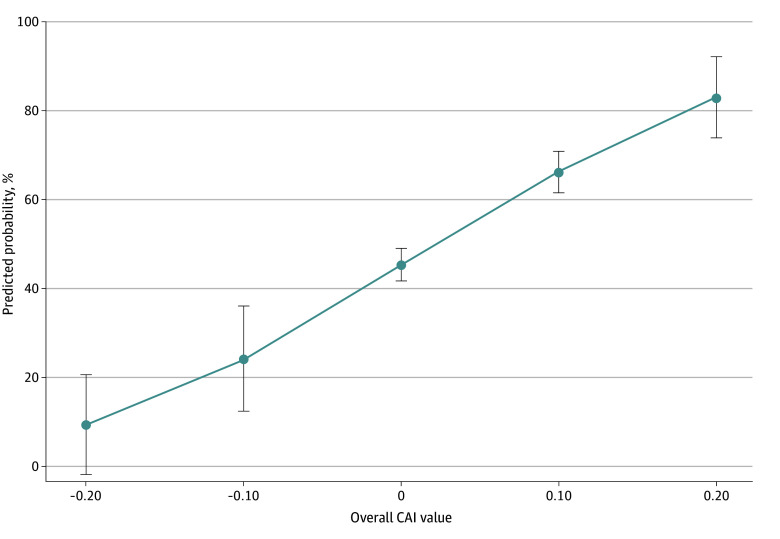
Dot Plot of Estimated Probability of Bonus Eligibility at Levels of Overall Categorical Adjustment Index (CAI) This figure presents the estimated probability and 95% CI of bonus eligibility at various levels of overall CAI value. These probabilities are estimated using the linear fixed effects panel regression model predicting by-year bonus eligibility (model 1). Actual CAI values range between −0.1 to 0.2, but the x-axis is slightly wider for symmetry and as a predicted value if CAI values were made even more negative.

**Figure 2.**
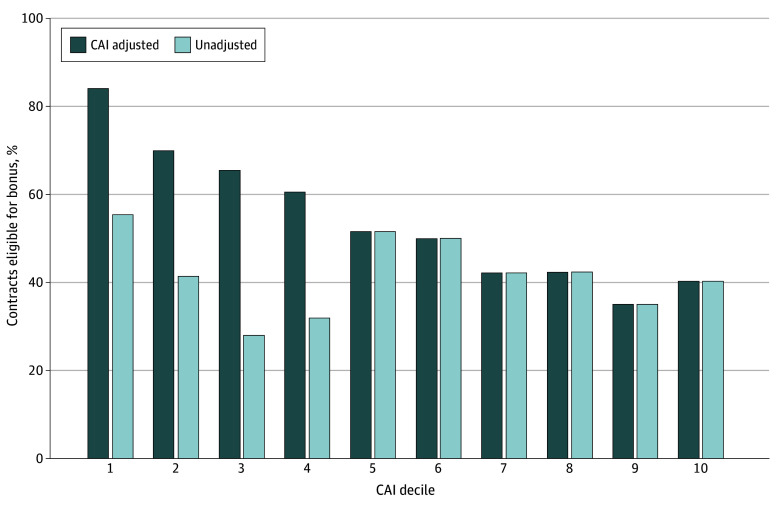
Bar Graph of Share of Medicare Advantage (MA) Contracts Eligible for Bonus Payments, by Categorical Adjustment Index (CAI) Decile and Adjustment Status, 2017–2025 Contracts are grouped into deciles based on the CAI, which ranks MA contracts by the share of enrollees with high social risk (recipients with low-income subsidy and/or beneficiaries with disabilities). CAI-adjusted values reflect bonus eligibility after applying the Centers for Medicaid & Medicare CAI policy, implemented beginning in 2017. Decile 1 represents contracts with the lowest social risk; decile 10, the highest.

eFigure 2 in Supplement 1 displays the distribution of overall CAI values across contract-years from 2017 to 2025. Figure 1 displays the estimated probability of bonus eligibility at all levels of overall CAI. Figure 2 displays the share of contract-year observations eligible for bonus payments before and after CAI adjustment across deciles of social risk. Unadjusted bonus eligibility decreased steadily with increasing social risk (approximately 50% in decile 1 vs 20% in decile 10). After CAI adjustment, eligibility increased across all deciles, increasing in decile 1 (to approximately 80%), but contracts in decile 10 remained substantially less likely to achieve 4 stars (approximately 40%).

In contract fixed-effects regressions with year fixed effects, each 0.10-unit increase in CAI was associated with a 0.16-point increase in probability of bonus eligibility (95% CI, 0.10 to 0.21; *P* < .001) (Table 3). Results were unchanged after adjustment for log enrollment (0.16; 95% CI, 0.10 to 0.21; *P* < .001). Using continuous star ratings, a 0.10-unit increase in CAI corresponded to a 0.18-point higher star rating (95% CI, 0.11 to 0.26; *P* < .001). Findings were similar in sensitivity analyses stratified by Part C and Part D star ratings (eTables 1-7 in Supplement 1).

**Table 3.** Association of CAI With Bonus Eligibility and Star Ratings, 2014–2025^a^

Statistic	Model 1: bonus eligibility^b^	Model 2: bonus eligibility + enrollment^b^	Model 3: star rating^b^
CAI value, point increase (95% CI)	0.16 (0.10-0.21)	0.16 (0.10-0.21)	0.18 (0.11-0.26)
Observations, No.	3566	3559	2624
Contracts, No.	339	339	337

Abbreviation: CAI, Categorical Adjustment Index.

^a^ Contract and year fixed effects; SEs clustered by contract. Coefficient is per 0.10-unit change in CAI (positive or negative).

^b^ Estimates are from contract fixed-effects regressions with year fixed effects; SEs clustered by contract. Model 1 includes CAI only; model 2 adds log(enrollment); model 3 uses continuous star rating (0–5) as the outcome. CAI is scaled per 0.10; values range –0.06 to 0.20, with negatives indicating downward adjustments.

## Discussion

Billions of dollars in annual bonus payments to MA plans are determined by a rating system that has long been criticized as unfair to plans serving populations at high risk.^15^ The MA star ratings program rewards plans that achieve high quality scores, but health plans with higher shares of enrollees with low income, disabilities, or dual eligibility have historically received lower ratings.^8^ The CAI was introduced to improve fairness in MA star ratings by adjusting for the share of enrollees with social risk factors. A recent study that examined the impact of CAI between 2017 and 2020 and found modest changes, with upward adjustments concentrated among contracts serving high proportions of enrollees with LIS or disabilities, and approximately one-quarter of such contracts qualifying for bonus payments as a direct result of CAI in 2020 alone.^16^

Our analysis quantifies not only the number of contracts shifted into bonus eligibility but also assessed the average marginal effect estimates for CAI in crossing the bonus threshold. In doing so, this study provides, to our knowledge, the first contract-level longitudinal evidence that each 0.10 increase in CAI value was associated with an approximate 0.16-point higher probability of bonus eligibility. These findings suggest that the CAI is associated with an increased probability that contracts near the 4-star threshold cross into bonus eligibility. However, our models include contract and year fixed effects, but cannot fully isolate CAI from other contemporaneous changes (eg, measure set revisions, cut-point recalibration, pandemic-era flexibilities). Star rating bonus payments have increased from roughly $3 billion in 2015 to more than $12 billion in 2025.^4^ While our analysis was not designed to generate precise fiscal estimates, these figures suggest that CAI-induced changes in bonus eligibility likely correspond to several billions of dollars annually.

Our findings raise questions about whether this form of social risk adjustment (ie, CAI) is adequate. The CAI applies a fixed value across all measures and years for a contract, which may not capture variation in performance challenges across different clinical domains or over time.^10^ Moreover, the adjustment is bounded in size, limiting its capacity to fully offset structural disadvantages in quality reporting or performance.^16^ As critiques of risk adjustment frameworks have argued, incorporating social risk into predictive models may improve statistical goals without delivering equitable outcomes.^17^ In the context of quality measurement, restoring parity in ratings may fall short of addressing disparities in care or access among populations with greater social risk factors. The adjustment is based only on low-income subsidy/dual eligibility and disability, excluding other important determinants of performance such as differences in social support, language, or neighborhood disadvantage.^18^ One potential direction would be to integrate validated measures such as the Area Deprivation Index, which accounts for neighborhood-level socioeconomic disadvantage and may better capture the structural determinants influencing enrollee health outcomes.^19^

Notably, contracts with the highest proportions of enrollees with LIS, dual eligibility, and disabilities received the largest CAI adjustments yet still tended to have the lowest underlying ratings and remained well below the 4-star threshold. This indicates that the CAI provides the greatest benefit to midperforming contracts, while offering limited practical benefit for the plans serving the most socially at-risk populations.^20^ Bonus payments support benefit generosity and plan competitiveness, and as a result, inequitable rating structures may indirectly limit the availability of enhanced benefits for beneficiaries in high-risk communities.^21^

These results also have implications for the ongoing CMS efforts to integrate social risk into star ratings. Beginning with the 2027 star ratings, the Excellent Health Outcomes for All (EHO4all) reward will provide incentives based on measure-level performance and improvement for enrollees with dual eligibility, LIS status, and disabilities while eliminating the existing reward factor that has historically boosted ratings for contracts with high and consistent performance.^22^ CMS has also indicated that additional factors, such as geography, may be incorporated into the EHO4all framework in future rulemaking. Unlike the CAI, which adjusts ratings uniformly based on enrollee mix, the EHO4all reward is linked to actual performance for underserved groups.

Our findings suggest that the CAI mainly helps midperforming contracts cross the bonus threshold. Contracts with the highest social risk receive the largest adjustments but rarely reach 4 stars. Our data cannot show whether their lower ratings reflect structural barriers, true differences in care quality, or both. Because contract-level ratings do not include measure-level detail, we cannot separate these explanations. For this reason, our results should not be interpreted as supporting broader social risk adjustment. Instead, they show that a uniform contract-level adjustment such as the CAI has limited ability to change bonus eligibility for the highest-risk contracts.

As CMS continues to modernize star ratings and other quality incentive programs, broad and static adjustments such as CAI may offer only partial solutions. Adjusting star ratings does not directly address disparities in care or access, meaning that even contracts receiving higher ratings and bonus payments as a result of CAI may continue to face persistent gaps in enrollee outcomes. Because the CAI raises ratings for the lowest-performing, highest-risk contracts but rarely by enough to move them across the 4-star bonus threshold, future approaches may need to go beyond uniform contract-level adjustments. Future reforms could adjust individual quality measures for social risk, place greater weight on measures that disproportionately affect disadvantaged groups, or compare plans against benchmarks tailored to their enrollee mix.^23,24^ These strategies may provide a framework for comparing plan performance fairly across diverse enrollee populations, while maintaining incentives for high-quality care delivery.

### Limitations

This study has several limitations. First, while we use a fixed-effects design to control for time-invariant differences across contracts, our results remain observational and cannot establish causal relationships. Second, we rely on publicly available data at the contract level and did not examine plan or individual-level variation in care or quality. Third, our models do not account for potential changes in contract behavior or coding practices in response to the CAI. Fourth, we approximated unadjusted ratings by subtracting the CAI from the rounded value rather than from the underlying continuous score. This conservative approach likely underestimates the true number of contracts whose bonus eligibility depended on CAI, as some contracts just below the threshold would have rounded up only after CAI was applied. Finally, while we document transitions in bonus eligibility, we do not assess the downstream effects of CAI on plan finances, benefit generosity, or enrollee experience.^25^

## Conclusions

This study found that the CAI expanded bonus eligibility in the star ratings program by increasing the probability that contracts just below the 4-star threshold crossed into bonus status. By contrast, contracts with the highest proportions of socially at-risk enrollees, though receiving the largest adjustments, remained well below the cutoff and experienced little change in bonus eligibility. Future research should assess whether alternative adjustment methods can create better comparisons of performance between MA contracts. Policymakers should consider whether refinements to the CAI are needed to ensure that star ratings better reflect true performance rather than underlying differences in enrollee risk.

## Notes

Supplement 1. **eFigure 1.** Sample inclusion flowchart **eFigure 2.** Distribution of overall CAI values across all years (2017-2025) **eTable 1.** Comparison of initial contract star ratings by pre-/post-CAI status **eTable 2.** Fixed effects regression predicting overall score (N=339) **eTable 3.** Fixed effects regression predicting Part C score (N=345) **eTable 4.** Fixed effects regression predicting Part D score (N=426) **eTable 5.** Contract characteristics by Part C bonus eligibility movement due to CAI (2014-2025) **eTable 6.** Contract characteristics by Part D bonus eligibility movement due to CAI (2014-2025) **eTable 7.** Fixed-effects panel regression predicting by-year Part C bonus eligibility (2014-2025)

Supplement 2.
Data Sharing Statement
