# Supplementary material for: Social Risk Adjustment and Bonus Eligibility in Medicare Advantage Star Ratings
Source: JAMA Health Forum. 2026 Mar 27;7(3):e260326. doi: 10.1001/jamahealthforum.2026.0326 (PMC13032144; doi:10.1001/jamahealthforum.2026.0326)
Supplement: Supplement 1. — eFigure 1. Sample inclusion flowchart eFigure 2. Distribution of overall CAI values across all years (2017-2025) eTable 1. Comparison of initial contract star ratings by pre-/post-CAI status eTable 2. Fixed effects regression predicting overall score (N=339) eTable 3. Fixed effects regression predicting Part C score (N=345) eTable 4. Fixed effects regression predicting Part D score (N=426) eTable 5. Contract characteristics by Part C bonus eligibility movement due to CAI (2014-2025) eTable 6. Contract characteristics by Part D bonus eligibility movement due to CAI (2014-2025) eTable 7. Fixed-effects panel regression predicting by-year Part C bonus eligibility (2014-2025) [file jamahealthforum-e260326-s001.pdf]

Supplemental Online Content

Anderson A, Satpathy-Horton R, Meiselbach MK. Social risk adjustment and bonus eligibility in Medicare Advantage star ratings. *JAMA Health Forum.* 2026;7(3):e260326. doi:10.1001/jamahealthforum.2026.0326

eFigure 1. Sample inclusion flowchart

eFigure 2. Distribution of overall CAI values across all years (2017-2025)

eTable 1. Comparison of initial contract star ratings by pre-/post-CAI status

eTable 2. Fixed effects regression predicting overall score (N=339)

eTable 3. Fixed effects regression predicting Part C score (N=345)

eTable 4. Fixed effects regression predicting Part D score (N=426)

eTable 5. Contract characteristics by Part C bonus eligibility movement due to CAI (2014-2025)

eTable 6. Contract characteristics by Part D bonus eligibility movement due to CAI (2014-2025)

eTable 7. Fixed-effects panel regression predicting by-year Part C bonus eligibility (2014-2025)

This supplemental material has been provided by the authors to give readers additional information about their work.

© 2026 Anderson A et al. *JAMA Health Forum*.

eFigure 1. Sample inclusion flowchart

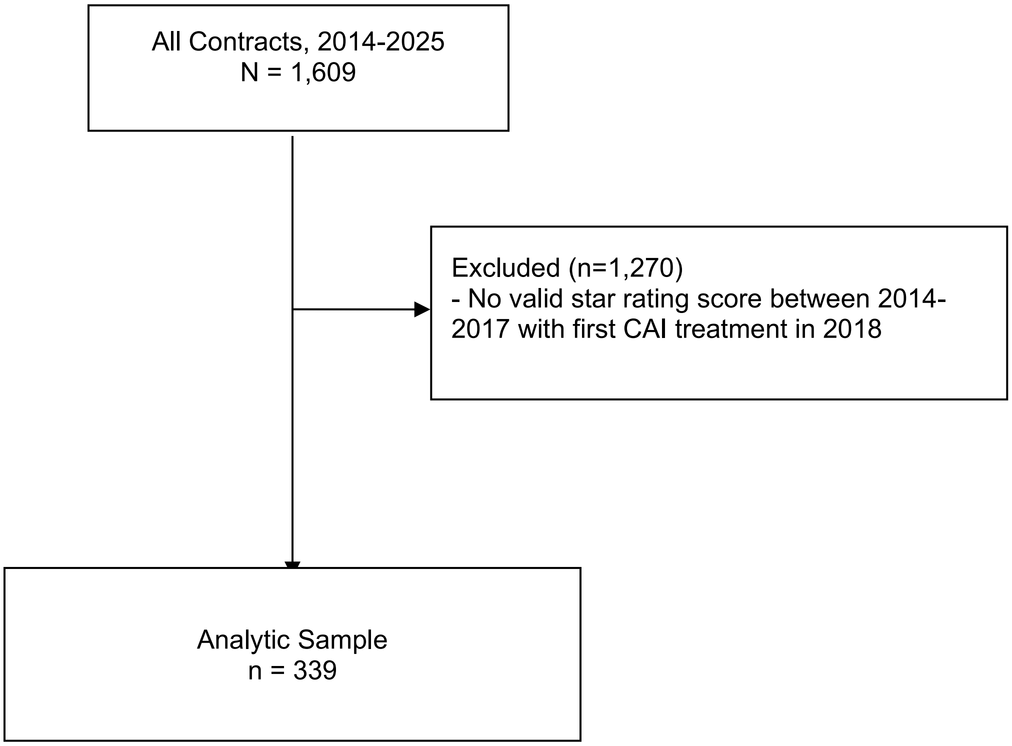

© 2026 Anderson A et al. *JAMA Health Forum*.

eFigure 2. Distribution of overall CAI values across all years (2017-2025)

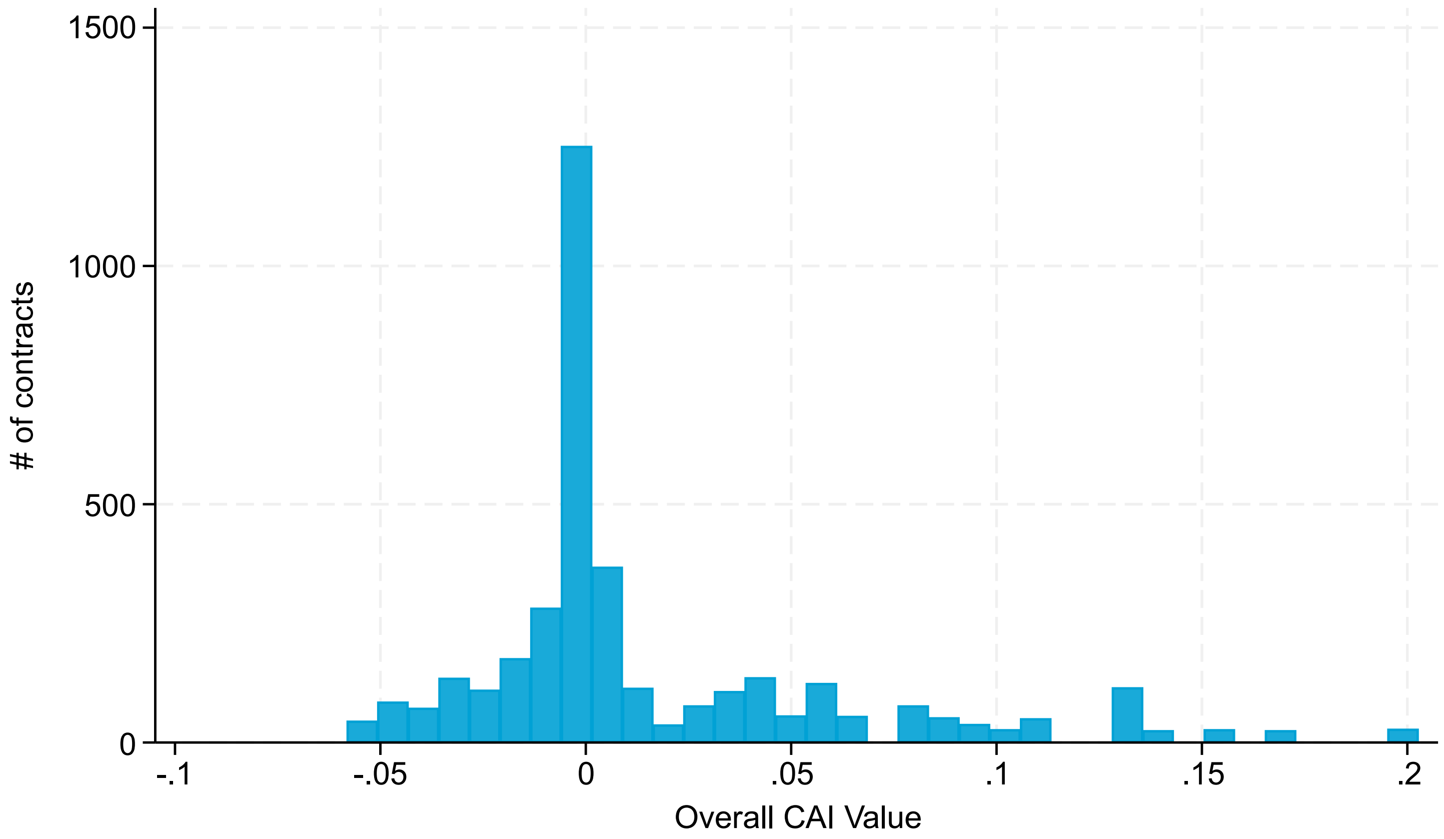

Notes: The figure above presents the distribution of overall CAI values across all years in the study period (2017-2025). The vertical axis represents the number of contracts that received a given value in any year. The sample used to generate this figure includes n=3,708 contract-year observations, representing n=339 distinct contracts. The sample is restricted to contracts which had at least one year of pre-CAI implementation data (2014-2016).

© 2026 Anderson A et al. *JAMA Health Forum*.

eTable 1. Comparison of initial contract star ratings by pre-/post-CAI status

	N	Mean	Std. Err	95% CI Lower	95% CI Upper	p-value
Pre-CAI	94	3.330	0.064	3.202	3.458	
Post-CAI	106	3.608	0.054	3.501	3.716	
combined	200	3.478	0.043	3.393	3.562	
diff		-0.279		-0.444	-0.113	0.001

Notes: Sample consists of N=200 contracts with an initial score between 2016-2018 or 2020-2022. “New contracts” defined using first valid score for contracts with 4 or less years elapsed since the contract effective date (i.e., pre-CAI contracts started between 2012-2014, post-CAI contracts started between 2016-2018).

© 2026 Anderson A et al. *JAMA Health Forum*.

eTable 2. Fixed effects regression predicting overall score (N=339)

star_overall_score	Coef.	Cluster Std. Err.	t-value	p-value	[95% Co	Interval]	Sig
**Overall CAI value**	1.912	0.315	6.06	0	1.292	2.533	***
**Year**							
2014	-0.058	0.029	-1.97	0.050	-0.116	0	*
2015	-0.067	0.028	-2.44	0.015	-0.121	-0.013	*
2016	0.028	0.022	1.28	0.201	-0.015	0.072	
2017 (ref)	0						
2018	-.002	0.023	-0.09	0.925	-.047	0.043	
2019	-0.044	0.026	-1.70	0.090	-0.095	0.007	
2020	0.054	0.026	2.06	0.040	0.002	0.106	*
2021	0.004	0.028	0.14	0.890	-0.051	0.058	
2022	0.362	0.027	13.19	0	0.308	0.416	***
2023	0.065	0.036	1.83	0.067	-.005	0.136	
2024	-.016	0.036	-0.44	0.660	-0.087	0.055	
2025	-0.088	0.037	-2.39	0.017	-0.160	-.016	*
**Constant**	3.792	0.02	191.53	0	3.753	3.831	***
Mean dependent var		3.85			SD dependent var		0.60
Overall R^2		0.13			Number of obs		3566.00
F-test		56.56			Prob > F		0.00
Within R^2		0.13			Between R^2		0.12

Notes: *** p<.001, ** p<.01, * p<.05

Notes: This table presents the results of a fixed-effects linear regression model predicting the overall star rating score using the overall CAI value and year, where the estimated variance is clustered by contract. The model includes N=339 contracts with a total of 3,566 observations. The sample is restricted to only contracts which had at least one non-missing overall star rating score prior to the implementation of the CAI in 2017.

© 2026 Anderson A et al. *JAMA Health Forum*.

eTable 3. Fixed effects regression predicting Part C score (N=345)

star_part_c_score	Coef.	Cluster Std. Err.	t-value	p-value	[95% Co	Interval]	Sig
**Part C CAI value**	1.524	0.492	3.10	0.002	0.556	2.492	**
**Year**							
2014	-0.038	0.030	-1.28	0.201	-0.097	0.021	
2015	-.027	0.028	-0.96	0.336	-0.082	0.028	
2016	-0.034	0.028	-1.20	0.229	-0.090	0.022	
2017 (ref)	0						
2018	0.011	0.025	0.43	0.667	-.039	0.061	
2019	-0.041	0.027	-1.51	0.131	-.094	0.012	
2020	0.018	0.028	0.62	0.533	-0.038	0.073	
2021	0.055	0.029	1.86	0.063	-.003	0.112	
2022	0.464	0.029	15.95	0	0.406	0.521	***
2023	0.142	0.037	3.85	0	0.069	0.214	***
2024	0.072	0.038	1.90	0.059	-.003	0.147	
2025	-0.042	0.037	-1.15	0.251	-0.115	0.030	
**Constant**	3.708	0.021	175.42	0	3.666	3.749	***
Mean dependent var		3.775			SD dependent var		0.610
Overall R^2		0.136			Number of obs		3626.000
F-test		65.784			Prob > F		0.000
Within R^2		0.136			Between R^2		0.013

Notes: *** p<.001, ** p<.01, * p<.05

Notes: This table presents the results of a fixed-effects linear regression model predicting the Part C star rating score using the Part C CAI value and year, where the estimated variance is clustered by contract. The model includes N=345 contracts with a total of 3,626 observations. The sample is restricted to only contracts which had at least one non-missing Part C star rating score prior to the implementation of the CAI in 2017.

© 2026 Anderson A et al. *JAMA Health Forum*.

eTable 4. Fixed effects regression predicting Part D score (N=426)

star_part_d_score	Coef.	Cluster Std. Err.	t-value	p-value	[95% Co	Interval]	Sig
**Part D Cai value**	0.971	0.167	5.82	0	0.643	1.299	***
**Year**							
2015	-0.161	0.031	-5.14	0	-0.223	-0.100	***
2016	0.094	0.029	3.27	0.001	0.037	0.151	**
2017 (ref)	0						
2018	0.037	0.029	1.26	0.207	-.02	0.094	
2019	0.005	0.031	0.18	0.861	-0.056	0.067	
2020	0.072	0.031	2.30	0.022	0.011	0.134	*
2021	-0.083	0.033	-2.51	0.012	-0.148	-0.018	*
2022	0.296	0.031	9.54	0	0.235	0.357	***
2023	-0.253	0.036	-7.02	0	-0.324	-0.182	***
2024	-0.132	0.037	-3.60	0	-0.204	-0.060	***
2025	-0.238	0.038	-6.18	0	-.313	-0.162	***
**Constant**	3.916	0.021	185.50	0	3.875	3.958	***
Mean dependent var		3.894			SD dependent var		0.646
Overall R^2		0.126			Number of obs		4094.000
F-test		64.244			Prob > F		0.000
Within R^2		0.126			Between R^2		0.120

Notes: *** p<.001, ** p<.01, * p<.05

Notes: This table presents the results of a fixed-effects linear regression model predicting the Part D star rating score using the Part D CAI value and year, where the estimated variance is clustered by contract. The model includes N=426 contracts with a total of 4,094 observations. The sample is restricted to only contracts which had at least one non-missing Part D star rating score prior to the implementation of the CAI in 2017.
Note that the Part D CAI value is a combined variable made by using both the MA-PD and PDP CAI values. These are mutually exclusive and whichever is not missing for a contract is used to generate the adjusted star rating score, so this allows for prediction of contracts which use either the MA-PD or PDP values in a single model.

© 2026 Anderson A et al. *JAMA Health Forum*.

eTable 5. Contract characteristics by Part C bonus eligibility movement due to CAI (2014-2025)

Variables	Never adjusted to bonus (n=218)	Ever adjusted to bonus (n=127)	Total (345)	P-value
**Mean Part C score**	3.50 (± 0.44)	4.10 (± 0.33)	3.72 (± 0.50)	<0.001
**Bonus movement events**	0.00 (± 0.00)	2.17 (± 1.17)	0.80 (± 1.27)	<0.001
**Primary geographic region**				<0.001
Northeast	37 (11.08%)	15 (4.49%)	52 (15.57%)	
Midwest	26 (7.78%)	23 (6.89%)	49 (14.67%)	
South	49 (14.67%)	20 (5.99%)	69 (20.66%)	
West	68 (20.36%)	22 (6.59%)	90 (26.95%)	
National	27 (8.08%)	47 (14.07%)	74 (22.16%)	
**Mean contract age (years)**	14.32 (± 6.85)	18.83 (± 8.29)	15.98 (± 7.71)	<0.001
**Years of data**	10.33 (± 2.91)	11.94 (± 0.29)	10.92 (± 2.45)	<0.001
**Mean Part C CAI value**	0.02 (± 0.02)	0.00 (± 0.01)	0.01 (± 0.02)	<0.001
**Mean minimum Part C CAI value**	0.00 (± 0.01)	-0.02 (± 0.01)	-0.01 (± 0.01)	<0.001
**Mean maximum Part C CAI value**	0.05 (± 0.05)	0.02 (± 0.03)	0.04 (± 0.04)	<0.001
**Mean % SNP enrollment**	0.51 (± 0.36)	0.20 (± 0.29)	0.41 (± 0.37)	<0.001
**Mean % MA-PD LIS enrollment**	44.64 (± 33.51)	18.38 (± 21.82)	34.91 (± 32.28)	<0.001
**# MA enrollees (1000s)**	6.72 (± 30.03)	24.45 (± 99.11)	15.01 (± 71.59)	0.069
**# Part D enrollees (1000s)**	45.56 (± 98.39)	82.08 (± 153.63)	59.09 (± 122.87)	0.008
**# overall enrollees (1000s)**	47.83 (± 111.82)	95.66 (± 197.68)	65.44 (± 150.78)	0.004

Notes: (1) CMS Star Ratings data (2014-2025). (2) P-values by t-test for continuous variables and Chi2 test for binary/categorical variables.

Notes: This table presents characteristics of all unique contracts based on if they were ever moved to bonus eligibility (>=4 stars) based on the Part C star rating score. Characteristics were collected from all years in which a contract provided data. The table includes only contracts which had at least one year of data pre-CAI implementation (2014-2016). Factor variables (region) are presented as the number of contracts (% of all contracts) in each group. Continuous variables are presented with the values of the mean (SD).

© 2026 Anderson A et al. *JAMA Health Forum*.

eTable 6. Contract characteristics by Part D bonus eligibility movement due to CAI (2014-2025)

Variables	Never adjusted to bonus (n=164)	Ever adjusted to bonus (n=262)	Total (426)	P-value
**Mean Part D score**	3.55 (± 0.45)	4.04 (± 0.39)	3.86 (± 0.48)	<0.001
**# years moved to bonus**	0.00 (± 0.00)	2.33 (± 1.42)	1.43 (± 1.59)	<0.001
**Primary geographic region**				<0.001
Northeast	25 (6.04%)	34 (8.21%)	59 (14.25%)	
Midwest	25 (6.04%)	32 (7.73%)	57 (13.77%)	
South	44 (10.63%)	37 (8.94%)	81 (19.57%)	
West	34 (8.21%)	57 (13.77%)	91 (21.98%)	
National	29 (7.00%)	97 (23.43%)	126 (30.43%)	
**Mean contract age (years)**	11.99 (± 6.45)	16.34 (± 7.68)	14.66 (± 7.53)	<0.001
**# years of data**	9.71 (± 3.23)	11.35 (± 1.67)	10.72 (± 2.52)	<0.001
**Mean Part D CAI value**	0.04 (± 0.05)	-0.02 (± 0.06)	0.00 (± 0.06)	<0.001
**Mean minimum Part D CAI value**	-0.02 (± 0.07)	-0.09 (± 0.11)	-0.06 (± 0.10)	<0.001
**Mean maximum Part D CAI value**	0.12 (± 0.10)	0.04 (± 0.07)	0.07 (± 0.09)	<0.001
**Mean % SNP enrollment**	0.56 (± 0.35)	0.32 (± 0.35)	0.43 (± 0.37)	<0.001
**Mean % MA-PD LIS enrollment**	53.60 (± 33.18)	23.88 (± 26.18)	35.48 (± 32.49)	<0.001
**Mean % PDP LIS enrollment**	30.57 (± 25.45)	3.15 (± 2.41)	12.64 (± 19.83)	<0.001
**# MA enrollees (1000s)**	5.43 (± 26.13)	17.84 (± 82.08)	14.41 (± 71.29)	0.253
**# Part D enrollees (1000s)**	145.22 (± 588.60)	80.46 (± 217.23)	105.39 (± 403.54)	0.107
**# overall enrollees (1000s)**	147.12 (± 589.51)	88.31 (± 237.19)	110.95 (± 410.68)	0.151

Notes: (1) CMS Star Ratings data (2014-2025). (2) P-values by t-test for continuous variables and Chi2 test for binary/categorical variables.

Notes: This table presents characteristics of all unique contracts based on if they were ever moved to bonus eligibility (>=4 stars) based on the Part D star rating score. Characteristics were collected from all years in which a contract provided data. The table includes only contracts which had at least one year of data pre-CAI implementation (2014-2016). Factor variables (region) are presented as the number of contracts (% of all contracts) in each group. Continuous variables are presented with the values of the mean (SD).

© 2026 Anderson A et al. *JAMA Health Forum*.

eTable 7. Fixed-effects panel regression predicting by-year Part C bonus eligibility (2014-2025)

Variables	Coef.	Cluster Std. Err.	t-value	p-value	[95% Co	Interval]	Sig
**Part C CAI Value**	0.384	0.517	0.74	0.459	-0.636	1.404	
**Overall enrollment (ln)**	0.038	0.022	1.72	0.086	-.005	0.082	
**MA-PD LIS enrollment %**	-.005	0.003	-1.70	0.091	-0.011	0.001	
**SNP enrollment %**	0.006	0.002	2.38	0.018	0.001	0.011	*
**Primary geographic region**							
Northeast	0.061	0.065	0.94	0.347	-0.067	0.190	
Midwest	-0.080	0.084	-0.96	0.339	-0.245	0.085	
South	-0.073	0.085	-0.85	0.394	-0.242	0.095	
West	-0.327	0.125	-2.62	0.01	-0.573	-0.080	**
National (ref)	0						
**Contract age (years)**	-.01	0.006	-1.90	0.059	-0.021	0	
**Year**							
2014	-0.066	0.042	-1.58	0.116	-0.149	0.017	
2015	-0.061	0.038	-1.61	0.109	-0.135	0.014	
2016 (ref)	0						
2017	0.057	0.033	1.75	0.082	-.007	0.121	
2018	0.043	0.039	1.11	0.268	-0.033	0.119	
2019	0.062	0.044	1.41	0.160	-0.025	0.148	
2020	0.116	0.043	2.68	0.008	0.031	0.202	**
2021	0.122	0.040	3.06	0.002	0.043	0.200	**
2022	0.344	0.036	9.55	0	0.273	0.416	***
2023	0.111	0.040	2.79	0.006	0.033	0.189	**
2024	0.091	0.038	2.42	0.016	0.017	0.165	*
2025 (omitted)							
**Constant**	0.291	0.270	1.08	0.283	-0.242	0.824	

Mean dependent var	0.448	SD dependent var	0.497
Overall R^2	0.089	Number of obs	1773.000
F-test	7.367	Prob > F	0.000
Within R^2	0.089	Between R^2	0.042

Notes: (1) CMS Star Ratings data (2014-2025). (2) *** p<.001, ** p<.01, * p<.05

Notes: This table presents the results of a fixed-effects panel linear regression model predicting by-year Part C bonus eligibility, clustering the variance within each contract. The model includes the following variables: Part C CAI value, overall enrollment (ln), percent

© 2026 Anderson A et al. *JAMA Health Forum*.

MA-PD LIS enrollment, percent SNP enrollment, primary geographic region, contract age, and time (as a categorical variable indicating the year). The model includes n=1,773 contract-year observations, which consists of a total of 217 unique contracts using complete case analysis.

© 2026 Anderson A et al. *JAMA Health Forum*.
