# Supplementary material for: Social Risk Adjustment and Bonus Eligibility in Medicare Advantage Star Ratings
Source: JAMA Health Forum. 2026 Mar 27;7(3):e260326. doi: 10.1001/jamahealthforum.2026.0326 (PMC13032144; doi:10.1001/jamahealthforum.2026.0326)
Supplement: Supplement 2. — Data Sharing Statement [file jamahealthforum-e260326-s002.pdf]

Data Sharing Statement

Anderson. Social Risk Adjustment and Bonus Eligibility in Medicare Advantage Star Ratings. *JAMA Health Forum*. Published March 27, 2026. doi:10.1001/jamahealthforum.2026.0326

Data
Data available: Yes
Data types: Data (not involving human participants)
How to access data: The data are publicly available.
When available: With publication

Supporting Documents
Document types: None

Additional Information
Who can access the data: Any data including code can be made available on request
Types of analyses: any purpose
Mechanisms of data availability: without investigator support
